# Dataset accompanying “Investigating the limits of spectroscopy for the estimation of foliar N and P in apple”: Hyperspectral reflectance, foliar nutrient concentrations and associated metadata

**DOI:** 10.1016/j.dib.2025.112343

**Published:** 2025-12-04

**Authors:** Cameron B. Cullinan, Alice N. Scomparin, Massimo Tagliavini, Katrin Janik

**Affiliations:** a Laimburg Research Centre, Laimburg 6, Auer/Ora, BZ 39040, South Tyrol, Italy; b Faculty of Agricultural, Environmental and Food Sciences, Free University of Bolzano, Piazza Università 1, BZ 39100, South Tyrol, Italy; c Leopold-Franzens-Universität Innsbruck, Innrain 52, Innsbruck, 6020, Austria

**Keywords:** Remote sensing, Plant nutrition, Chemometrics, Automation, Precision agriculture, Sustainable Spectra, Spectroradiometer

## Abstract

This dataset was generated to support research investigating the use of hyperspectral reflectance for the estimation of foliar nitrogen (N) and phosphorus (P) concentrations in apple (*Malus domestica*) trees. This article and the dataset it describes accompany an original research article submitted to *Computers and Electronics in Agriculture* entitled “Investigating the limits of spectroscopy for the estimation of foliar N and P in apple” [1]. Data were collected from a controlled potted experiment involving 150 ‘Golden Delicious’ apple trees grown under varying nutrient supply regimes, including full nutrient supply, nitrogen- and phosphorus-deficient treatments, and trees infected with ‘*Candidatus* Phytoplasma mali’. The experiment was conducted over the 2023 growing season at the Laimburg Research Centre in South Tyrol, Italy. All data and the accompanying code for its analysis is freely available in the associated GitHub repository [2].

Spectral data were collected using the Spectral Evolution SR-3500 field spectroradiometer with an attached leaf clip, producing high-resolution hyperspectral reflectance profiles (350–2500 nm) from the adaxial surface of fully expanded leaves. A total of 1189 leaf spectra were recorded and were matched to chemically analysed leaf samples. Corresponding foliar N and P concentrations (and others) were determined through laboratory analysis using the Dumas combustion method for nitrogen and ICP-OES following acid digestion for phosphorus. The dataset includes metadata detailing tree treatments, sampling dates, infection status, and shoot growth metrics. Additionally, R scripts used for data processing, spectral pre-treatment (including multiplicative scatter correction and Savitzky-Golay derivatives), feature selection (VIP and mRMR), and model development are provided.

The dataset is suitable for reuse in the development and benchmarking of spectral models for nutrient estimation, especially in the context of field-based or remote sensing applications in horticulture. Its wide range of foliar nutrient values, inclusion of multiple physiological stresses, and detailed documentation make it a valuable resource for researchers working in precision agriculture, plant phenotyping, chemometrics, and hyperspectral data analysis.

Specifications Table

**Table:** 

Subject	Biology
Specific subject area	Hyperspectral spectroscopy-based estimation of foliar nitrogen (N) and phosphorus (P) in apple trees*.*
Type of data	Table, Spectral Data (.sed files), Graph, Figure, R scripts Raw, Analysed, Filtered, Processed.
Data collection	Spectral Evolution SR-3500 spectroradiometer with leaf clip; foliar nutrient content via Dumas method and ICP-OES.
Data source location	Laimburg Research Centre, South Tyrol, Italy, 46.3827° N, 11.2877° E.
Data accessibility	Repository name: Github Data identification number: DOI 10.5281/zenodo.15600557 Direct URL to data: https://github.com/HyperspectralCameron/Investigating-the-Limits-of-Spectroscopy-for-the-Estimation-of-Foliar-N-and-P-in-Apple.gitInstructions for accessing these data: All data and code are publicly available through the GitHub repository listed above. The repository includes raw spectral files (.sed), metadata files, and R scripts for pre-processing, modelling, and visualisation. No registration or authentication is required. Users may clone or download the repository directly.
Related research article	Submitted as Cullinan, C., Scomparin, A., Janik, K., & Tagliavini, M. (2025). Investigating the Limits of Spectroscopy for the Estimation of Foliar N and P in Apple Computers and Electronics in Agriculture*.*

## 1 Value of the Data

• Provides a large dataset of leaf-level hyperspectral reflectance (350–2500 nm) from apple trees under controlled nutrient conditions.
• Includes matched foliar N and P concentrations across biologically relevant ranges.
• Useful for researchers in plant phenotyping, agronomy, precision agriculture, and remote sensing model development.
• Enables testing and benchmarking of spectral pre-processing methods, feature selection algorithms (e.g. mRMR, VIP), and chemometric modelling techniques (e.g. PLSR).

## 2 Background

Full-range UV–VIS-NIR spectroscopy offers a non-destructive alternative to traditional lab-based nutrient analyses, but questions remain regarding its robustness in the face of confounding or perturbing factors. This dataset was compiled to investigate the use of hyperspectral reflectance spectroscopy to estimate foliar nitrogen (N) and phosphorus (P) concentrations in apple (*Malus domestica*) leaves in the presence of external perturbations, namely P and N soil deficiencies and infections with ‘Ca. Phytoplasma mali’. The data article supports the original research article, “Investigating the Limits of Spectroscopy for the Estimation of Foliar N and P in Apple,” by making the complete dataset publicly available. The analyses presented in this article make use of only small subset of those potentially applicable. By making the data and the code that analyses it available, we provide the opportunity for further investigation. By including spectral data, laboratory analyses and metadata we encourage the further methodological development and independent exploration of the data for, amongst others, model development, feature selection techniques and sensor designs for precision agriculture.

## 3 Data Description

The data described in this article is all found in the home directory of the GitHub repository under the following structure:

**/Scripts**

**/Scripts/R_script.R**

For spectral pre-processing (MSC, Savitzky-Golay), PLSR modelling, feature selection (VIP, mRMR), and visualisation. Within the script is code that incorporates all the appropriate data within the other files into a single dataset. All file paths are set relative to this file. Hence, if the structure of the files is not changed, R_script.R should be ready to run as is (after extracting all compressed files.)

**/Scripts/R_script_hsdar.R**

The original analysis presented in the original research article was performed using functions contained in the R package *hsdar* as done in R_script_hsdar.R. *hsdar* has, however, since been removed from CRAN and has *rgdal* as a dependency, which cannot run on R 4.3 and later. R_script. R provides the same analysis that does not rely on *hsdar*. R_script_hsdar.R is included for the sake of transparency and reproducibility. Should you wish to run this code, you can copy the *hsdar* package into the directory in which R accesses downloaded libraries. You will need to install its dependencies (*rgdal* will only work on older versions of R).

**/Scripts/training_trees**

A list of trees in .RDS format. Data from these trees were used to train the models presented in the research article accompanying this data. Read by R_script.R.

**/Scripts/test_trees**

A list of trees in .RDS format. Data from these trees were used to test the models presented in the research article accompanying this data. Read by R_script.R.

**/hsdar and /rgdal.7z**

R packages that are no longer available within CRAN. To replicate the analysis performed by R_script_hsdar.R, these packages should be copied and pasted into the library repository used by R.

**/Experimental_information.xlsx**

Metadata relating the treatment applied to each tree. Given for each tree is the:

• TreeID: a unique number used to identify each tree
• Treatment: which treatment was applied to each tree i.e. cont – Control trees, APP – trees infected with ‘Ca. P. mali’, N – trees experiencing a severe N deficiency, P - trees experiencing a severe P deficiency, LN - trees experiencing a moderate N deficiency and MoP - trees experiencing a severe P deficiency.
• PCR: the infection status of the tree (where appropriate) as determined by polymerase chain reaction (PCR) assay of the roots. Infected trees (producing a positive PCR result) are designated by “I”, while unifected, PCR-negative trees are designated by “U”.
• Congruence: designates whether a tree’s infection status is congruent with the treatment it received, i.e. whether control trees were really uninfected and whether trees that were grafted with infected scions produced a positive PCR result. Where appropriate, trees whose treatment was incongruent with the treatment they were given are removed from some analyses (infections did not establish in all the trees that were grafted with infected scions).
• PPM: key to files produced by PCR assay (for administrative purposes and of little relevance to the analysis and removed by R_script.R)

**/ShootGrowth2023.xlsx**

Shoot growth measured at the end of the growing season. The data consists of a:

• TreeID: a unique identifier corresponding those within /experimental_information.xlsx
• Shoot Order: empty vector (not used or relevant within this dataset)
• ShootType: whether the shoot measured was the apical shoot (denoted as p) or a lateral (denoted a).
• Length: Total length of the shoot in cm

**/LICENCE**

Copyright terms of use.

**/README.md**

Short description of the data repository.

**/Raw_Data.zip**

Raw data used by R_script.R. **NB extract all files before running R_script.R**

Contents after unzipping:

**/Raw_Data/Analysis Results**

Foliar nutrient concentrations of N and P in .xlsx format, one file each for the N and P (and others) foliar concentrations obtained from leaves sampled at three different dates. Table 1 summarises the data in this folder. R_script.R combines data, removes unnecessary information and renames data with more intuitive names (in English).

1) Foliar N (Raw_Data/Analysis Results/Nitrogen):
  a) As determined using the TruSpec Micro System(LECO Corporation, St. Joseph, MI, USA) and the Dumas method (DIN EN ISO 16,634–1:2008)
  b) Column names:
    i) **Codice**: unique code used by lab to identify samples, not relevant to data analysis, removed by R_script.R.
    ii) **Data**: the date that samples were collected
    iii) **DenominazioneAppezzamento:** Sample name supplied with the sample. A combination of the Tree ID and the treatment that tree had received (corresponding to (/Experimental_information.xlsx)
    iv) **Varietà:** Apple cultivar sammpled (all Golden Delicious)
    v) **GiornoFioritura Data:** Date of full bloom (all 04/04/2023)
    vi) **N-Blatt@Elementaranalyse-DIN@** **%Stickstoff (N) (** **%):** Foliar N Concentration in percent (on the basis of weight per dry weight)
2) Foliar P (Raw_Data/Analysis Results/Phosphorus):
  a) As determined by microwave assisted acid digestion (ultraWAVE Single Reaction Chamber technology; Milestone Srl; Italy) and determination with inductively coupled plasma optical emission spectroscopy (ICP-OES 720 Axial, Agilent Technologies, USA; EPA 3052:1996 + EPA 6010D:2018)
  b) Column names:
    i) Codice – GiornoFioritura Data: as for foliar N
    ii) P@HNO3-H2O2-Aufschluss/MW/ICP-OES@ %Phosphor (P) ( %): Foliar P concentration in percent (wt/dry wt)
    iii) Data also includes the foliar concentrations of other nutrients determined by ICP-OES (each is designated by their standard symbol on the periodic table). The original research paper accompanying this article investigated the estimation of foliar N and P under differential supply of these nutrients. Since all trees were supplied similar amounts of the other nutrients, these nutrients were not analysed.

**Table 1:** Summary of the data contained within the Raw_Data directory. Given is the nutrient that was measured by laboratory methods, the date on which the leaves that were submitted were sampled, the number of spectra that were taken of the leaves submitted for sampling, the mean ± standard deviation and range of the foliar concentration of all the samples taken on that date as well as the range across all the samples.

Nutrient	Date	Count	Mean	Nutrient Range ( % wt/wt)	
N	30-May-23	146	2.24±0.41	1.17 - 2.87	0.83 - 2.8
	30-Jun-23	145	1.87±0.42	0.83 - 2.43	
	10-Jul-23	144	1.84±0.43	0.84 - 2.59	
P	2-May-23	48	0.24±0.04	0.16 - 0.31	0.06 - 0.37
	9-Jun-23	141	0.2 ± 0.06	0.09 - 0.37	
	10-Jul-23	144	0.2 ± 0.06	0.06 - 0.31	
Total	-	768	-	-	-

**/Raw_Data/SED files**

**Fig. 1:**
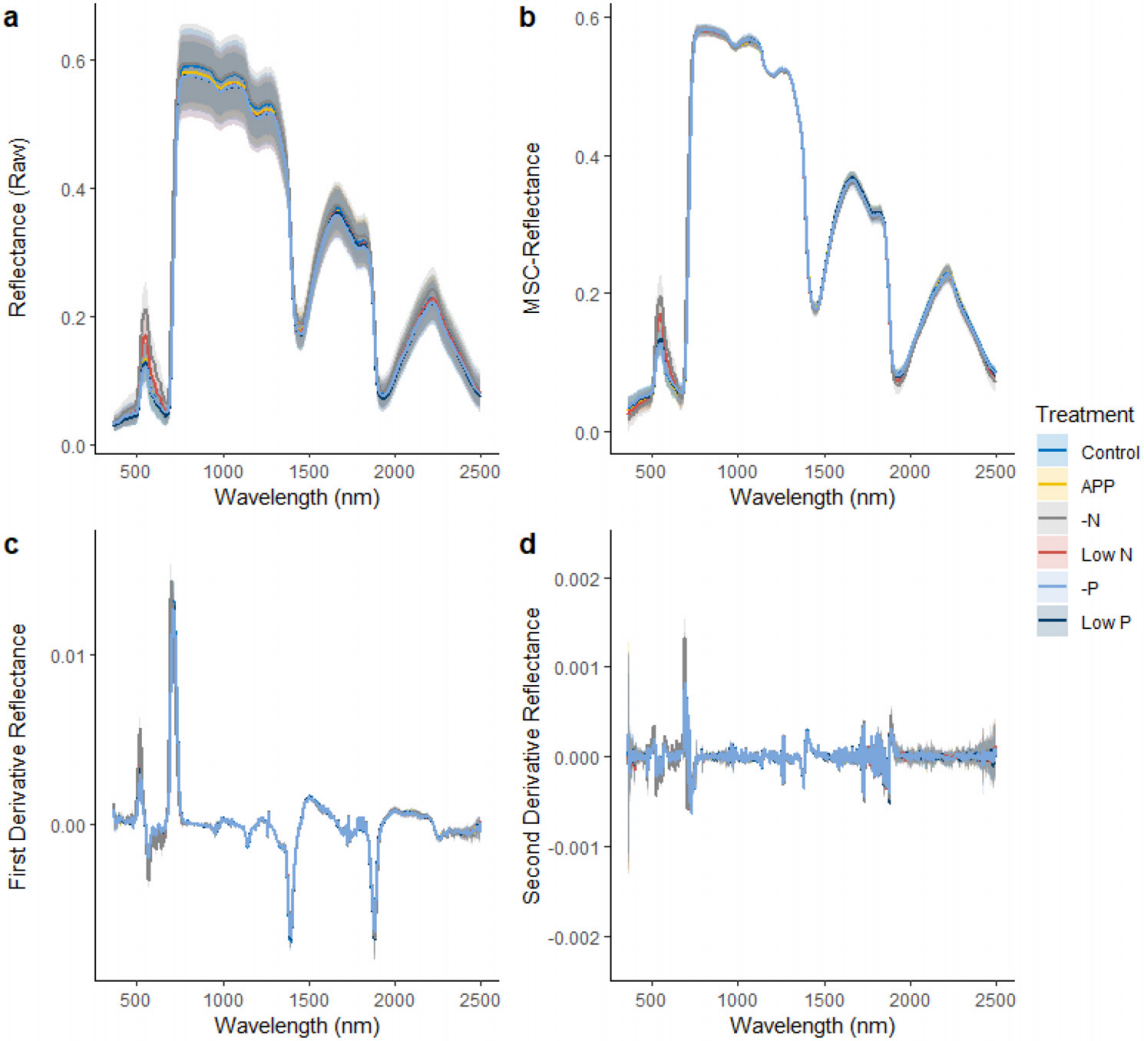
a) Raw spectra generated from the raw .sed files using the code contained in the R script (b) Multiplicative Scatter Corrected (MSC)-spectra and (c) first and (d) second Savitzky-Golay derivative spectra generated from the raw spectra using the code contained in the R script. Lines and shaded regions represent the means ± the standard deviations at each wavelength of each treatment.

Spectral Data: 1189 spectra of individual leaves (in .sed format), covering 350–2500 nm in folders according to the date in which they were collected. Read and integrated with other data by R_script.R. Fig. 1 shows the raw spectra and the spectra after applying correction algorithms (as performed in R_script.R)

**/Raw_Data/Excel trees**

Key relating each spectrum to a sample submitted for chemical analysis (one file exists for the leaves from which spectra were obtained and subsequently submitted for analysis for each date). The code to perform this is contained in R_script.R

Table 1 summarises the data contained within the raw data directory after aggregating spectra and associating each one with a corresponding foliar nutrient concentration.

## 4 Experimental Design, Materials and Methods

The experimental design that was used to generate this data is as described in the accompanying research article. Below are a summary and some further elaborations:

### 4.1 Experimental design

**Fig. 2:**
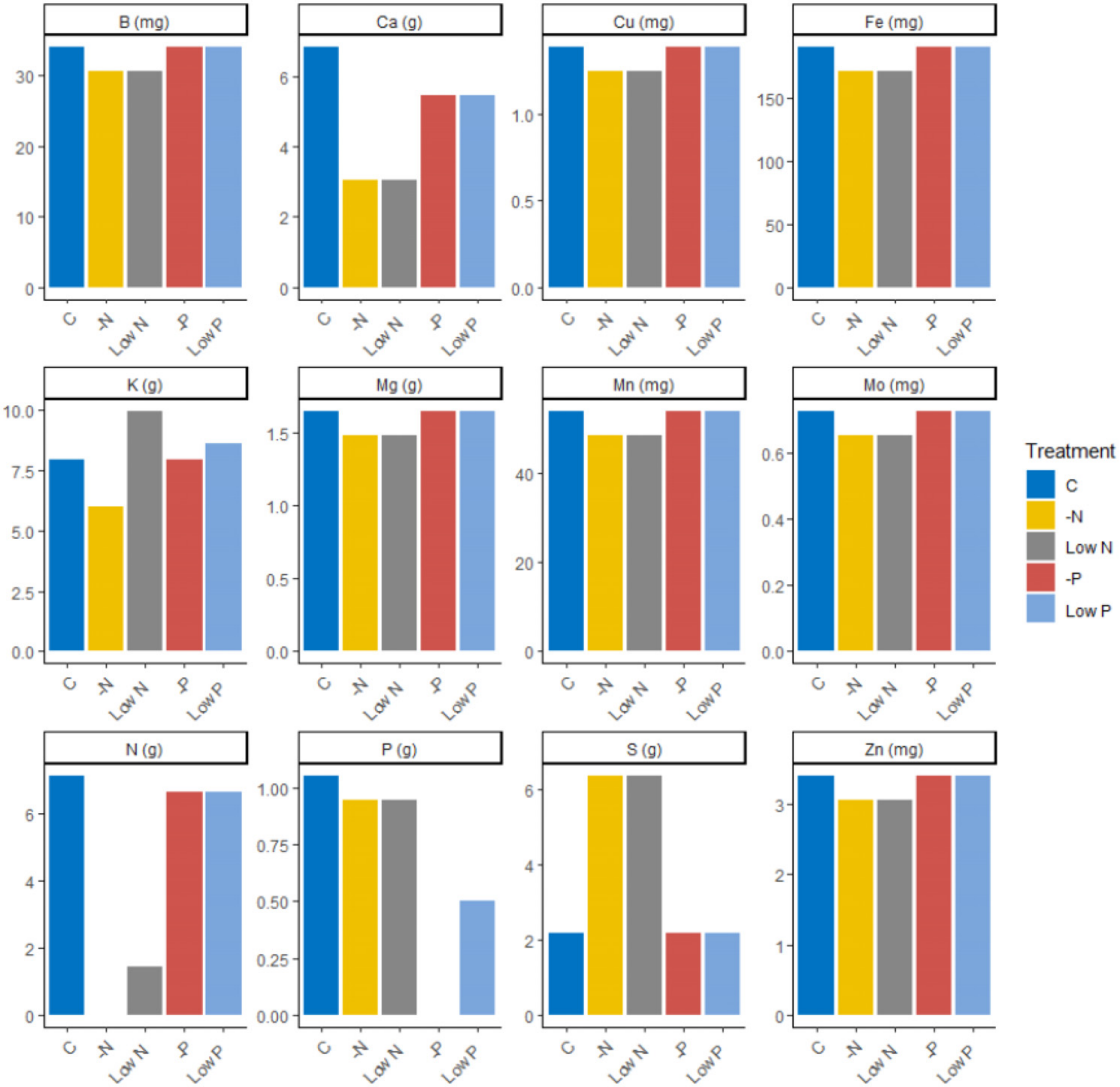
Estimated total supply of each plant mineral nutrient given to each plant within a given treatment in 2022 and 2023. Nutrient solutions were added approximately weekly. Upon observation of symptoms of micronutrient deficiencies in 2022, the amount of each micronutrient supplied to each tree was doubled.

A potted trial (*n* = 150) was established in a foil tunnel at the Laimburg Research Centre, South Tyrol, Italy. Two-year-old trees were planted in a low nutrient, 80:20 sand-peat medium in May 2022. To obtain good variation in the foliar concentrations of N and P, nutrient solutions with different amounts of each nutrient were added to different sets of the trees. Some trees were supplied with either a complete, an N-free or a P-free solution as described in Hoagland and Arnon [3] but doubling the amount of each micronutrient supplied in the standard solution. The remaining trees were supplied with either nutrient solution containing 20 % of the total N given to the controls (supplied as KNO3) or nutrient solution containing 50 % of the total P supplied to the controls (supplied as KH_2_PO_4_; see Fig. 2). Nutrient solutions were supplied approximately weekly from March to August in 2022 and 2023. To facilitate the application of nutrient solutions (so that the risk of human error was minimised), trees were arranged such that rows consisted of approximately four trees of the same treatment. Rows of any given treatment were randomly arranged in the foil tunnel.

Of the 70 trees supplied with complete solution, 40 were infected with *ˈCa*. P. mali ˈ by grafting six scions (three each in May and August 2022), confirmed to be infected by PCR assay as described in Barthel et al. [4]. At the same time, the same number of uninfected scions were grafted into each of the control trees. After grafting in August, a mixture of gibberellic acid (GA3) and the cytokinin, 6-Benzyladenine, was applied to the trees to facilitate the graft union (Promalin, Valent Biosciences, USA). Table 2 summarises the treatments applied to the potted trees, the names by which each is referred to from here on out and the number of replicates in each treatment.

**Table 2:** Summary of the treatments applied to potted trees from which spectra and foliar samples were taken. Provided are the names by which each treatment is referred to throughout the article, how each treatment was established, the number of replicates within each treatment and the estimated total amount of N (g) and P (g) supplied to each tree over 2022 and 2023.

Treatment Class Name	Conditions	*n =* 150	N (g)	P (g)
Control	Full nutrient Solution Grafted with uninfected scions	30	10.5	1.55
APP	Full nutrient Solution Grafted with infected scions	40	10.5	1.55
-N	N-free nutrient solution Not grafted	20	0	1.39
Low N	20 % N solution (compared to Control) Not grafted	20	2.1	1.39
-P	P-free nutrient solution Not grafted	20	9.8	0
Low P	50 % P solution (compared to control) Not grafted	20	9.8	0.74

After transplanting each tree in 2022 the trees experienced substantial shock. In addition to this, each treatment required some time to manifest physiologically within the trees (i.e. to allow for APP infections to establish and allow mineral reserves within the trees to deplete within the appropriate trees). Therefore, only data obtained in 2023 are supplied and used in subsequent data analysis. Phytosanitary measures were applied for the control of powdery mildew (Podosphaera leucotrich Ellis & Everh.; E.S. Salmon), woolly apple aphid (Eriosoma lanigerum Hausmann), codling moth (Cydia pomonella Linnaeus), leaf miner (Lyonetia clerkella Linnaeus, Leucoptera malifoliella O. Costa), apple scab (Venturia inaequalis Cooke), European red mite (Panonychus ulmi Koch).

### 4.2 Shoot growth measurement, leaf sample collection, chemical laboratory analysis

Shoot growth was measured at the end of the growing season in November 2023. To do this the length of each shoot which had grown throughout the preceding season (i.e., the softwood shoot growth) of each tree was measured and summed to get the total. All fruitlets in 2023 were removed as early as possible to maximise resource allocation to vegetative growth.

Samples for nutrient determination by chemical analysis were collected at five sampling dates throughout the growing season (two timepoints for the collection of samples submitted for N analysis, two timepoints for the collection of samples submitted for P analysis and one in which samples for both were collected). Samples were taken from the fully expanded leaves, located in the central parts of the shoots. For the determination of N, 0.2 g of dried material were required which could be supplied by one leaf. For P, however, 0.3 g were required and so two leaves were required for each sample. In total, 447 and 411 leaf samples were submitted for laboratory analysis of N and P, respectively.

To prepare each sample, each leaf was first cleaned and had its midribs and petioles cut off and the leaves were packed tightly inside a 2.0 ml reaction tube (Eppendorf, Hamburg, Germany). The leaves were dried in the reaction tubes by placing them in a drying oven at 60 °C overnight. Following this, two 3 mm Tungsten Carbide (Qiagen, Hilden, Germany) beads were added to each tube, and the samples were milled at 30 Hz for six minutes using the TissueLyser II (Qiagen, Hilden, Germany). The powdered samples were then sent to the lab for the chemical determination of foliar N and P concentrations. The TruSpec Micro System and the Dumas method (DIN EN ISO 16,634–1:2008) were employed for the determination of foliar N concentrations. For the determination of foliar P concentrations, microwave assisted acid digestion (ultraWAVE Single Reaction Chamber technology; Milestone Srl; Italy) and determination with inductively coupled plasma optical emission spectroscopy (ICP-OES 720 Axial, Agilent Technologies, USA; EPA 3052:1996 + EPA 6010D:2018) were used.

Some of the lab-determined nutrient concentrations were extraordinarily high. These were assumed to have been the result of a technical error in their measurements. R_script.R removes these outliers. To do this, outliers are conservatively identified as being more the three inter-quartile ranges above and below the means of their respective groups (a threshold of 1.5 interquartile ranges is more commonly used). A total of five outliers is identified, which are then removed from the dataset and all subsequent analyses.

R_scipt.R also analyses total shoot growth and the foliar concentrations of N and P according to the treatment the tree received. The means of the total shoot growth of each treatment as well as those of the foliar nutrient concentrations of each treatment at each date are compared by ANOVA followed by post-hoc *t*-tests, with Holm’s multiple comparison correction, comparing each mean to the relevant control-mean. The validity of the assumptions of residual normality and homoscedasticity are assessed visually using normal quantile-quantile plots and plots of residuals against fitted values, respectively.

### 4.3 Hyperspectral data acquisition

Spectra were acquired from the exact leaves that were submitted for chemical analysis (i.e., fully expanded leaves). In May and June, spectra were taken from the leaves while still attached to the trees. In July, when the conditions in the tunnel were too hot and humid to be suitable for us to spend long periods of time inside, the leaves were removed from the tree and the spectra were taken immediately afterwards. Hyperspectral profiles of leaves were obtained using the Spectral Evolution SR-3500 spectroradiometer with the accompanying leaf clip for direct leaf measurements (Spectral Evolution, USA). The conical-conical reflectance factor [5], against the white spectralon reference incorporated into the leaf clip, was obtained from the adaxial surface of each leaf at each sampling date by contact measurement. Dark references were taken automatically by the device before each scan. Spectra were taken from the centres of each leaf (midveins were not avoided but unusual blemishes were). White references were taken approximately every 10 min.

### 4.4 Data processing and analysis

#### 4.4.1 Preprocessing

Spectral data were acquired using the DARWin™ SP Data Acquisition Software (Spectral Evolution, USA) supplied with the spectroradiometer and are located in “/Raw_Data/SED files”. All subsequent data analysis is performed by R_script.R. Spectra are imported from the SED files with spectrolab [6] and assigned the metadata obtained manually during their acquisition (using the files in “/Raw_Data/Excel trees” and “/Experimental_information.xlsx”).

For the estimation of phosphorus measured by ICP-OES, where two leaves needed to be pooled for the chemical analysis, the spectrum corresponding to each sample is calculated as either the arithmetic, geometric and harmonic means of the two spectra taken of each of the leaves combined into one sample (only the arithmetic mean is used in subsequent analysis) . A total of 1189 spectra were taken (770 after aggregating the appropriate spectra). Unfortunately, a technical malfunction in the computer being used to acquire the data occurred during the collection of data on May 2 where 130 spectra were lost (hence the discrepancy between leaf samples analysed and the number of spectra acquired).

Outliers in the spectra (or those showing an atypical shape for that of vegetation) are identified according to their angular distances from the mean spectrum using the spectral angle mapper algorithm [7]. Spectra having an angle with the mean spectra more than three standard deviations from the mean angle of all spectra are classified as outliers. The spectra corresponding to these outliers are then visualised (for human confirmation) and excluded from further analysis. The use of spectral angle theoretically isolates outliers based on disproportionality with a typical spectrum which, from our experience, is a more reliable indicator that a technical malfunction occurred during the acquisition of a spectrum. Only two spectral outliers were removed. In total, using the thresholds as described above, 7 data points (5 outliers in lab determined nutrient concentration and 2 spectral outliers) are removed, representing <0.1 % of the dataset. Thresholds can be adjusted to identify outliers more or less conservatively.

Spectra are pre-processed by either multiplicative scatter correction (MSC) or the first or second Savitsky-Golay derivatives (see Fig. 1). MSC is performed using the “pls” package [8]. In the analysis presented in the accompanying the original research paper, the Savitsky-Golay derivatives, were produced using with the *hsdar* package with a smoothing window of 5 [9]. In addition to this, *hsdar* conveniently provides a set of functions for calculating 115 pre-established spectral vegetation indices (SVIs). A data set of these 115 SVIs were thus also generated from the full-range spectra and assessed along with the other pre-processed spectra sets. For the formulation and references to the original work of each index the reader is referred to Lehnert et al. [9] and https://rdrr.io/cran/hsdar/f/inst/doc/References.pdf.

Frustratingly, *hsdar*, has since been removed from CRAN. For reproducibility, the package along with its dependency, *rgdal* [10] have been included in the Github repository and can be copied into the directory in which R accesses downloaded libraries (*rgdal* will have to be unzipped first) which should permit R_script_hsdar.R to run. *rgdal* will not run on R 4.3 and later and so R_script_hsdar.R will require older versions of R to run. To get around these problems, the original R script (R_script_hsdar.R) has been adapted (into R_script.R) to perform the same functions but without the use of *hsdar*. Savitzky-Golay filters are performed using signal (signal developers, 2023) and SVIs are calculated using a newly defined function after the one supplied in *hsdar*.

After processing, R_scipt.R plots the spectra such that the mean spectrum ± the standard deviation of each treatment is shown.

### 4.5 Development of PLSR models

PLSR was used to build predictive models. PLSR is a statistical method used to predict a set of dependent variables from a set of independent variables by extracting a few orthogonal factors, known as latent variables (LVs), that explain the most variance in both sets [11]. These latent variables are linear combinations of the original variables, designed to maximize the covariance between the predictors and responses. These latent variables are then sequentially added to a linear regression model in decreasing order of importance until a negligible improvement or deterioration in predictive performance is observed (as determined through calibration or cross validation). This incremental addition of LVs during cross-validation helps prevent overfitting and ensures that the model generalizes well to new data and so is well suited to spectral data which consists of highly multivariate, correlated data.

R_script.R divides the data into training and test sets by tree, such that spectra from one tree are present exclusively in either the training set or the test set to avoid information carryover from the training set to the test set. Trees were divided randomly at a 80:20 training to test ratio.

PLSR models are trained and tested on data from the whole growing period as well as the data from each month individually. On the pooled data from the full vegetative period, using the package, *caret* [12], thrice repeated, 10-fold cross validation is used to tune the optimal combination of preprocessing technique (MSC and derivatives) and the number of latent variables (up to 50). The data are scaled and centred internally by the training function. The optimal combination is selected as that which produced the lowest mean root mean squared error (RMSE) across all the repeated folds. The significance of the relationships between predicted and measured test set values are assessed by a linear mixed effect model using the packages lme4 [13] and lmerTest [14] (Satterthwaite's degrees of freedom method), with the predicted value as a fixed effect and random intercepts for data collected from each tree nested within a given treatment.

The preprocessing procedures that produced the best results on the full vegetative growth period (i.e. MSC and the arithmetic mean; see Results), are then used to train each of the models for the prediction of N and P using the data acquired on each of the sampling dates alone (to assess whether there is a dependency on the date of sampling). Similarly, models are also trained and tested on the sets of SVIs at each individual date.

### 4.6 Extraction of wavelength importance from PLSR models and recursive feature elimination

The importance of each wavelength for each model is extracted from the full MSC-spectra and SVI models trained on the data from the full vegetative period using the variable importance in projection (VIP) and the minimum redundancy, maximum relevance (mRMR) algorithms using the package *plsVarSel* [15]. Based on these results, a recursive feature elimination is performed in which thrice repeated, 10-fold cross validated models are trained on subsets of the full feature sets such that features are recursively eliminated in increasing order of importance.

## Limitations

The major limitation regarding this data is that it has been acquired from a potted trial under controlled conditions and only in a single cultivar of apple of uniform age (2-year-old Golden Delicious). It remains to be seen whether the models trained upon this data will perform as well in the field where a whole host of external factors that could interfere with them are to be expected. Likewise, the use of the models trained from this dataset may not be appropriate for trees of different ages and cultivars.

The models trained using R_script.R make use of only PLSR algorithms (because they are efficient and interpretable). There is notable potential for the development of models using other algorithms. Thus, the authors encourage the use of this dataset for further exploration and development of models using spectroscopic methods.

## Ethics Statement

The authors confirm that they have read and follow the ethical requirements for publication in *Data in Brief*. The current work does not involve human subjects, animal experiments, or any data collected from social media platforms.

## CRediT Author Statement

**Cameron Cullinan:** Conceptualization, Methodology, Software, Formal analysis, Investigation, Data Curation, Writing - Original Draft, Writing - Review & Editing, Visualization, Project administration. **Alice N. Scomparin:** Conceptualization, Methodology, Investigation, Data Curation. **Massimo Tagliavini:** Conceptualization, Methodology, Writing - Review & Editing, Supervision, Project administration. **Katrin Janik :** Conceptualization, Methodology, Resources, Writing - Review & Editing, Supervision, Project administration, Funding acquisition

## Data Availability

Dataset accompanying “Investigating the Limits of Spectroscopy for the Estimation of Foliar N and P in Apple”: Hyperspectral reflectance, foliar nutrient concentrations and associated metadata (Original data)
